# Editorial: Telemedicine During and Beyond COVID-19

**DOI:** 10.3389/fpubh.2021.662617

**Published:** 2021-03-16

**Authors:** Sonu Bhaskar, Alma Nurtazina, Shikha Mittoo, Maciej Banach, Robert Weissert

**Affiliations:** ^1^Pandemic Health System REsilience PROGRAM (REPROGRAM) Global, Sydney, NSW, Australia; ^2^Department of Neurology, Liverpool Hospital and South Western Sydney Local Health District, Sydney, NSW, Australia; ^3^Neurovascular Imaging Laboratory & NSW Brain Clot Bank, Ingham Institute for Applied Medical Research, Sydney, NSW, Australia; ^4^Department of Epidemiology and Biostatistics, Semey Medical University, Semey, Kazakhstan; ^5^Department of Rheumatology, University Health Network and The University of Toronto, Toronto, ON, Canada; ^6^Polish Mother's Memorial Hospital Research Institute, Lodz, Poland; ^7^Cardiovascular Research Centre, University of Zielona Góra, Zielona Gora, Poland; ^8^Department of Hypertension, Medical University of Lodz, Lodz, Poland; ^9^Department of Neurology, University of Regensburg, Regensburg, Germany

**Keywords:** COVID-19, digital health, telemedicine, public health, policy

Telemedicine has been at the mainstay of patient care by offsetting the decline in outpatient visits during the Coronavirus disease 2019 (COVID-19) while providing critical patient continuity and limiting exposure to health systems and healthcare workers (1). However, there are concerns that the decline in outpatient visits has not been entirely offset by telemedicine, which may have consequences beyond the COVID-19 pandemic (2). The current Research Topic, “*Telemedicine during and beyond COVID-19*,” presents a collection of articles on telemedicine during and beyond COVID-19. The COVID-19 pandemic is causing an unprecedented public health crisis impacting healthcare systems, healthcare workers, and communities. The COVID-19 Pandemic Health System REsilience PROGRAM (REPROGRAM) consortium is an independent not-for-profit think-tank of international healthcare physicians, researchers, and policymakers formed to champion the safety of healthcare workers, policy development, and advocacy for global pandemic preparedness and action with a focus on advocacy and building capacity in under-resourced settings [Bhaskar et al. (a), (3)].

In addition to presenting an overview on disparities in telemedicine globally [Bhaskar et al. (a)], and across various medical specialties [Bhaskar et al. (b)], including teleneurology in Sub-Saharan Africa (Adebayo et al.), it also explores the current and potential applications of technologies such as artificial intelligence and robotics in designing futuristic telemedicine (Bhaskar, Bradley, Sakhamuri et al.). A study by Sinha et al. report on the implementation and evaluation of a video visit program at an academic practice in New York (USA) demonstrating promise for telemedicine in the primary care settings during COVID-19.

During the initial phase of the pandemic, acute shortages in global medical supplies were reported. An article in the current topic presents a model to profile critical medical stockpiles and improve the medical supply chain through the use of technologies such as advanced analytics and blockchain (Bhaskar, Tan et al.). Indeed, the provision of adequate medical supplies such as personal protective equipments (PPEs) and mechanical ventilators are warranted to mitigate the risks to healthcare workers and health systems and build capacity for future infectious disease outbreaks. Moreover, COVID-19 disrupted traditional medical education and training (Sharma and Bhaskar). This has led to the integration of telemedicine into medical education and training. The telemedicine enabled medical education system may continue beyond COVID-19 especially in providing mental health support to medical students in general, and especially those from vulnerable backgrounds.

On another tangent, Lehner et al. from Germany, share their experiences on an online blog in assisting psychiatric patients, who have been rendered increasingly vulnerable due to social isolation and loneliness due to lockdown measures, during COVID-19. The ongoing and future mental health toll due to COVID-19 calls for increased attention, where telepsychiatry has a potential role to play as it has been received favorably by patients during various phases of COVID-19 lockdown (4). Merianos et al. present perspectives on the use of telemedicine toward tobacco cessation and prevention in rural areas during COVID-19. Seifert et al. provide key recommendations on mitigating the digital divide in delivering telemedicine to elderly patients in long-term care facilities, which have been severely impacted during the COVID-19. Interestingly, given the focus on telerehabilitation, apropos to which Stasolla et al. present an assistive-technologies based approach in supporting patients with neurological conditions and communication difficulties. COVID-19 has adversely impacted the provision and access of healthcare services to chronic disease patients (5), including those with acute and chronic neurological conditions (3, 6).

In conclusion, despite the broadening scope of telemedicine and rapid roll-out during the COVID-19, systemic issues such as organizational readiness, including digital maturity, licensing, regulatory hurdles, reimbursements, ability to be used by all groups, including the oldest and those with disabilities, infrastructural issues and geographical and digital disparities in telemedicine adoption warrant urgent attention [Bhaskar et al. (a); Bhaskar et al. (b)]. Future efforts should pivot around increasing telemedicine access and provision to those from marginalized communities and under-resourced settings (7). Telemedicine could play an important role in expanding the outreach to remote areas and those from vulnerable backgrounds (8, 9), as well as to developed and under-developed nations carrying a disproportionate burden of vulnerable communities [Bhaskar et al. (a)]. This should be complemented with efforts to standardize telemedicine care and/or workflows using common tools for the clinical examination which could improve telemedicine practice and quality of care (10). The increasing use and expansion of telemedicine are likely to persist beyond the COVID-19; therefore, building equitable telehealth systems should be central to our preparedness and public health response for the future, especially in the advent of a future pandemic.

## Author's Note

The COVID-19 pandemic is causing an unprecedented public health crisis impacting healthcare systems, healthcare workers, and communities. The COVID-19 Pandemic Health System REsilience PROGRAM (REPROGRAM) consortium is formed to champion the safety of healthcare workers, policy development, and advocacy for global pandemic preparedness and action.

## Author Contributions

All authors discussed the results and recommendations and contributed to the final manuscript.

## Conflict of Interest

The authors declare that the research was conducted in the absence of any commercial or financial relationships that could be construed as a potential conflict of interest.
